# Comparative Genomic Analysis of Clonal Variants of a *Spirabiliibacterium* Lineage Recovered from a Chicken in the United States

**DOI:** 10.3390/microorganisms14091953

**Published:** 2026-09-03

**Authors:** Lakshmi T. Sunkara, John Reddy Peasari, Diane Davis, Radhika Kakani, Tanit Kasantikul, Rob Harbert, Maurice Byukusenge

**Affiliations:** 1 Clemson Veterinary Diagnostic Center, Livestock Poultry Health, Clemson University, Columbia, SC 29229, USA; jpeasar@clemson.edu (J.R.P.);; 2 Veterinary Diagnostic Laboratory, Michigan State University, Lansing, MI 48910, USA; 3 United Kingdom Animal Diagnostic Laboratory, Oxford Nanopore Technologies, Inc., Exeter OX4 4DQ, UK; 4 Department of Veterinary and Biomedical Sciences, Pennsylvania State University, University Park, PA 16802, USA

**Keywords:** chickens, *Pasteurellaceae*, long-read WGS, *Spirabiliibacterium*, USA

## Abstract

The family *Pasteurellaceae* includes pathogenic and opportunistic bacteria affecting poultry, yet taxonomic resolution remains challenging due to phenotypic heterogeneity, limited genomic representation, and the poor performance of routine diagnostic systems for uncommon taxa. At the Clemson Veterinary Diagnostic Center, a Gram-negative bacterium was isolated from a backyard hen. The isolate produced two distinct colony morphotypes with identical biochemical profiles. They were misidentified as *Sphingomonas paucimobilis* by VITEK^®^ 2, while MALDI-TOF MS failed to identify them. Oxford Nanopore long-read sequencing with PacBio HiFi polishing generated near-complete chromosome-level assemblies. Comparative genomic analyses demonstrated that morphotypes CVDC-smooth and CVDC-rough represent clonal variants of a distinct *Spirabiliibacterium* lineage sharing 93% average nucleotide identity (ANI) and 52% digital DNA–DNA hybridization (dDDH) with the closest relative, *Spirabiliibacterium mucosae*. Copy-number variation within a tandemly duplicated ~20 kb genomic region may contribute to differences in colony morphology. Family-wide analysis identified lineage-associated gene patterns and a limited number of VFDB-matched virulence genes in the CVDC isolates. This study identifies a distinct lineage within the genus *Spirabiliibacterium*, reports the first isolation and chromosome-level genome assemblies of a *Spirabiliibacterium* lineage from a chicken in the United States, and highlights the limitations of routine diagnostic methods for identifying uncommon bacterial taxa.

## 1. Introduction

The family *Pasteurellaceae* comprises Gram-negative, non-motile, non-spore-forming coccobacilli, or short rods, that are often pleomorphic and possess relatively small genomes [1,2]. These fastidious bacteria grow under aerobic or facultatively anaerobic conditions at approximately 37 °C and commonly colonize the upper respiratory and reproductive tracts of birds and other animals as commensals, although some species act as opportunistic pathogens under conditions of stress, immunosuppression, or coinfection [1,3,4]. In poultry, infections caused by members of this family range from acute septicemia to chronic respiratory or reproductive disease and may occasionally involve joints or other tissues [1,3,5]. Among avian *Pasteurellaceae*, *Pasteurella multocida* and *Gallibacterium anatis* are well-recognized pathogens that encode virulence factors contributing to colonization and disease [5,6,7]. Although many members exhibit host-associated ecological preferences, their pathogenic potential and evolutionary relationships remain incompletely understood [1,8,9].

Traditional diagnostic methods, such as biochemical testing and use of VITEK^®^ 2, provide limited resolution for closely related taxa, including members of the *Pasteurellaceae* [10,11,12]. Although MALDI-TOF mass spectrometry enables rapid bacterial identification in many diagnostic settings, it may fail to reliably classify rare, divergent, or poorly represented species in reference databases [13,14]. Phenotypic heterogeneity further complicates identification, particularly when colony morphology varies, despite similar metabolic profiles; such variation may arise from underlying genomic differences not detected by routine biochemical testing [15,16]. Consequently, bacterial classification increasingly relies on molecular approaches, including 16S rRNA gene sequencing and whole-genome sequencing (WGS) [1,10,17]. The value of WGS for resolving the identity of uncommon bacterial isolates that cannot be reliably identified by routine diagnostic methods has also been demonstrated in other bacterial genera [18]. Long-read sequencing technologies, including Oxford Nanopore Technologies (ONT) and Pacific Biosciences (PacBio), enable complete genome assembly, resolution of repetitive regions, and robust comparative genomic and phylogenetic analyses [19,20,21]. Despite these advances, extensive genomic diversity and highly open pangenomes continue to complicate taxonomic resolution within the family *Pasteurellaceae*, resulting in ongoing revisions [1,17,22]. This taxonomic uncertainty is exemplified by several Bisgaard taxa that could not be reliably classified based on their phenotypic characteristics and have been reported from a variety of avian species and rodents, with or without associated clinical disease [1,4,10].

Bisgaard taxon 14, initially characterized by its phenotypic and biochemical characteristics, has been associated with respiratory disease and fowl cholera-like disease in poultry [9]. Over time, numerous *Pasteurella*-like isolates have been assigned to this taxon, although some have remained unidentified using conventional diagnostic methods [4,11]. Bisgaard taxon 14 was subsequently reclassified as *Spirabiliibacterium mucosae* (*S. mucosae*) based on phylogenetic, genomic, and phenotypic analysis [10]. Publicly available genomic resources for *S. mucosae* remain limited, including a fragmented multi-contig assembly for the type strain 20609/3ᵀ (GCF_014884965.1, NCBI RefSeq) and Illumina short-read data for the TN_CUL_2021 (SRR19976462; NCBI Sequence Read Archive) [10,17]. Genomic analyses of these isolates identified limited virulence and antimicrobial resistance (AMR) genes, suggesting their potential relevance to avian health [17]. However, the limited availability of complete genome assemblies continues to hinder comparative genomic analyses, taxonomic resolution, and the accurate identification of uncommon clinical and veterinary isolates.

In 2022, two colony morphotypes, designated CVDC-smooth and CVDC-rough, were recovered from a bacterial isolate obtained from a backyard chicken submitted to the Clemson Veterinary Diagnostic Center (CVDC). Conventional biochemical and MALDI-TOF mass spectrometry methods failed to reliably identify the isolates. Subsequent whole-genome sequencing and comparative genomic analyses suggested that CVDC-smooth and CVDC-rough represent clonal variants of a genomically distinct *Spirabiliibacterium* lineage closely related to *S. mucosae*. These findings prompted further genomic and phenotypic characterization to clarify their taxonomic placement and potential biological significance.

The objectives of this study were to identify an unclassified bacterial isolate recovered from a chicken, characterize its genomic features, compare it with related members of the family *Pasteurellaceae*, and provide high-quality genomic references for the understudied genus *Spirabiliibacterium*. To our knowledge, this work presents the first chromosome-level genome assemblies for the genus *Spirabiliibacterium*, documents the first isolation of a *Spirabiliibacterium* lineage from a chicken in the United States, and expands the genomic resources available for future comparative, evolutionary, and diagnostic investigations of this poorly characterized lineage.

## 2. Materials and Methods

### 2.1. Clinical History and Routine Diagnostic Testing

In 2022, an 8-year-old Dominique hen from a backyard flock of six birds was submitted to CVDC, Columbia, South Carolina, USA, with a reported 1-year history of progressive partial paralysis, weight loss, and wing lesions. Because the etiology of the clinical disease was unknown at presentation, a complete necropsy and routine diagnostic investigation were performed to evaluate potential infectious agents. The diagnostic workflow included gross and histopathologic examination, screening for viruses and bacteria, and subsequent characterization of recovered bacterial isolates. Given the chronic clinical presentation and the need to exclude important infectious diseases of poultry associated with neurologic and/or neoplastic disease, uncut, formalin-fixed, paraffin-embedded (FFPE) tissue sections were submitted to the Michigan State University Veterinary Diagnostic Laboratory for immunohistochemistry (IHC) to rule out Marek’s disease virus (MDV), avian leukosis virus (ALV), and reticuloendotheliosis virus (REV). These agents were evaluated as part of the differential diagnostic investigation because the underlying disease etiology was unknown.

To investigate the potential role of bacteria in disease, swabs were collected from multiple tissues, including the liver, and from lesions identified at necropsy, and cultured on 5% sheep blood agar (Hardy Diagnostics, Santa Maria, CA, USA) at 37 °C with 5% CO_2_. Bacterial isolates recovered from culture were initially evaluated by Gram staining and basic biochemical assays. After the isolate was identified as Gram-negative, antimicrobial susceptibility testing (AST) was performed using the Kirby–Bauer disk diffusion method as part of the routine diagnostic bacteriology workflow at CVDC. The antimicrobial panel included β-lactams, aminoglycosides, tetracyclines, trimethoprim–sulfamethoxazole, macrolides, rifamycin, and sulfonamides (BD Diagnostics, Sparks Glencoe, MD, USA). In the absence of species-specific interpretive criteria for *Spirabiliibacterium*, NCCLS M31-A2 criteria were used as surrogate reference criteria for applicable antimicrobials. Streptomycin, for which corresponding criteria were not available in the NCCLS standard used, was evaluated using the diagnostic laboratory’s reference inhibition-zone criteria. Because validated breakpoints are unavailable for *Spirabiliibacterium*, these interpretations were considered descriptive rather than formal susceptibility classifications.

Further identification of the Gram-negative liver isolate was attempted using the VITEK^®^ 2 system (bioMérieux, Morrisville, NC, USA) and MALDI-TOF MS with the Bruker MALDI Biotyper Sirius system (Bruker, Saugus, MA, USA) with the BDAL-12438 Library Module (2023), date of access: February 2025; however, the isolate could not be reliably identified by either method. To further characterize this unidentified bacterial isolate, incubation was extended to 72 h, resulting in the appearance of two distinct colony morphotypes (CVDC-smooth and CVDC-rough) on blood agar. Additionally, the two morphotypes were cultured on MacConkey and chocolate agar to evaluate their growth on alternative media. These morphotypes were subcultured separately and selected for whole-genome sequencing to resolve their taxonomic identity and further investigate their genomic characteristics.

### 2.2. DNA Extraction and Sequencing

Genomic DNA was extracted from CVDC-smooth and CVDC-rough isolates using the Monarch^®^ Genomic DNA Purification Kit (New England Biolabs, Ipswich, MA, USA). Sequencing libraries were prepared using the ONT SQK-LSK114 ligation kit (Oxford Nanopore Technologies, Oxford, UK), and sequenced on a GridION (Oxford Nanopore Technologies, Oxford, UK) platform with FLO-MIN114 flow cells (one flow cell per isolate) for approximately 72 h following the manufacturer’s instructions. In parallel, both isolates were sequenced using PacBio HiFi technology at the University of Delaware DNA Sequencing and Genotyping Center by submitting bacterial isolates on 5% sheep blood agar slants.

### 2.3. Basecalling, Genome Assembly and Annotation, Taxonomic Identification

Oxford Nanopore (ONT) POD5 files were base-called using Dorado in super-accuracy mode. The resulting BAM files were converted to FASTQ using SAMtools [23,24], filtered with NanoFilt (minimum Q score 9) [25] and quality-checked with SeqKit [26]. No filters were applied for PacBio HiFi reads. Initial genome assembly attempts using Flye and the Nextflow EPI2ME wf-bacterial de novo assembly workflow failed to generate genome assemblies, likely due to the large ONT read dataset. Therefore, ONT and PacBio HiFi reads were assembled using AutoCycler [27], a de novo genome assembly pipeline incorporating read subsampling and multiple assemblers. The ONT assemblies of both isolates were initially polished by self-alignment to ONT reads using the Nextflow EPI2ME wf-bacterial-genome workflow in reference and isolate modes. Final polishing was performed with NextPolish [28], using PacBio HiFi reads, which provide higher per-base accuracy than ONT long reads, and enable more reliable detection of small insertions and deletions and variant fractions. Read-to-assembly consistency for CVDC-smooth and CVDC-rough was assessed using SAMtools with Nextflow EPI2ME-generated self-alignments. Genome circularization was confirmed by visual inspection of assembly graphs in Bandage [29], whereas assembly completeness and uniform read coverage were evaluated by mapping sequencing reads and inspecting coverage profiles in the Integrative Genomics Viewer (IGV) [30] prior to downstream analyses.

Assembly base-level accuracy and completeness were assessed with Merqury (k-mer size 21) [31]. Genome size and GC content were calculated using QUAST [32]. Genome completeness was assessed using BUSCO with the *Pasteurellales*_odb12 dataset [33]. Genome annotation was performed with Prokka [34]. Taxonomic identification was performed using the web-based Basic Local Alignment Search Tool for nucleotides (BLASTn (core_nt), [35], PubMLST, [36], GTDB-Tk v2.3.2 (via KBase), [37,38], and Kraken2 [39]. Genome assemblies of CVDC-smooth and CVDC-rough were also aligned to the genomes of *S. mucosae* 20609/3T and TN_CUL_2021 using Minimap2, and alignment statistics were calculated with SAMtools to evaluate genomic similarity and species-level relatedness [23,24,40].

### 2.4. Phylogenetics, Average Nucleotide Identity (ANI), and Genome-to-Genome Distance Analysis

Phylogenetic analysis was performed on 28 genomes, including 22 representative *Pasteurellaceae* genomes, 3 *Spirabiliibacterium* genomes from NCBI, CVDC-smooth and CVDC-rough isolates and *S. mucosae* strain TN_CUL_2021 (kindly provided by the authors) [17]. Multiple sequence alignments were generated using REALPHY v1.12 [41], with *Spirabiliibacterium* genomes as references. Phylogenetic trees were constructed with IQ-TREE v2.3.6, [42] under the GTR+G nucleotide substitution model, with branch support assessed using 1000 ultrafast bootstrap replicates, and were visualized using a custom Python (version: 3.13.14) script. Accession numbers for all genomes were provided in the Supplementary Materials (Table S1).

Average nucleotide identity (ANI) between CVDC-smooth, CVDC-rough, *S. mucosae* type strain 20609/3ᵀ, and TN_CUL_2021 was calculated using FastANI [43]. Digital DNA–DNA hybridization (dDDH) values were calculated using the Genome-to-Genome Distance Calculator (GGDC) with BLAST+ local alignment (Formula 2) [44]. Commonly accepted species-level genomic thresholds of ≥95% ANI and ≥70% dDDH were used to assess genomic relatedness [45,46].

### 2.5. SNP Distance Analysis

Pairwise single-nucleotide polymorphism (SNP) distances were calculated using SNP-dists v0.8.2 [47]. Whole-genome alignments were generated with REALPHY [41], using the CVDC-smooth, CVDC-rough, and publicly available *Spirabiliibacterium* genomes used as reference sequences. The resulting merged alignment was processed with SNP-dists using default parameters to produce a pairwise SNP distance matrix. Reported SNP distances represent single-nucleotide substitutions identified within genomic regions shared across all aligned genomes.

### 2.6. Pan-Genome, Virulence, and AMR Gene Analysis

Pan-genome analysis of 28 genomes, including CVDC-smooth and CVDC-rough, four *Spirabiliibacterium* genomes, and 22 *Pasteurellaceae* genomes, was performed using Roary v3.13.0 with a 95% BLASTp identity threshold [48]. The broader *Pasteurellaceae* dataset was included to provide a family-level comparative framework for evaluating gene-content patterns associated with *Spirabiliibacterium* and the CVDC isolates relative to other members of the family. The resulting gene presence–absence matrix was analyzed with Scoary2 to identify lineage-associated genes [49]. Default parameters were used unless otherwise specified. Core, soft-core, shell, and cloud gene categories were defined based on Roary output.

Virulence genes in the 28 genomes were screened against the Virulence Factor Database (VFDB) [50] and visualized using a custom Python (version: 3.13.14) script. AMR genes in CVDC-smooth and CVDC-rough were screened using ResFinder and the Comprehensive Antibiotic Resistance Database (CARD) via ABRicate [51,52]. A minimum threshold of ≥70% sequence identity and ≥80% coverage was applied for both virulence and AMR gene detection.

### 2.7. Variant Calling and Tandem Repeat Analysis

Genomic differences between the CVDC-smooth and CVDC-rough isolates were assessed using the BV-BRC Variation Analysis pipeline, which integrates BWA-MEM for read mapping, FreeBayes for variant calling, and SnpEff for functional annotation [53]. For this analysis, reads from CVDC-rough were aligned to the CVDC-smooth genome. PacBio HiFi reads were processed through the BV-BRC pipeline, while Oxford Nanopore Technologies (ONT) reads were analyzed using the Nextflow EPI2ME wf-bacterial-genome workflow. Variant profiles from both platforms were highly consistent; therefore, PacBio HiFi–derived variants were used for downstream analyses due to their higher per-base accuracy. Structural variants and tandem-repeat copy-number differences between CVDC-smooth and CVDC-rough were assessed by visualizing Minimap2 alignments in IGV and examining repeat junctions, read lengths, read coverage, and mapping percentage [40,54]. The repeat structures were further evaluated by performing BLASTn comparisons between CVDC-smooth and CVDC-rough, *S. mucosae* type strain 20609/3T (GCF_014884965.1) and TN_CUL_2021 [35].

### 2.8. Computational Analysis and Data Summary

All data analyses were conducted on the Palmetto 2 high-performance computing cluster (Clemson University, Columbia, SC, USA) using the SLURM scheduler, except for web-based tools [55].

Genome assemblies and raw sequencing reads generated in this study have been deposited in the National Center for Biotechnology Information (NCBI) under BioProject accessions PRJNA1447504 and PRJNA1448032. The isolates CVDC-smooth and CVDC-rough are associated with BioSample accessions SAMN56998712 and SAMN56998713, respectively. The accession numbers for genomes were JCCHDS000000000 for the CVDC-rough isolate and JCCHDT000000000 for the CVDC-smooth isolate. 16S rRNA gene sequences were extracted from the genome assemblies and deposited in the NCBI GenBank database under accession numbers PZ459834 and PZ459835. All software tools, versions, and parameters used in this study are provided in Supplementary Table S2.

## 3. Results

### 3.1. Clinical Findings

The bird was alive but weak on presentation, responsive to external stimuli, and in lateral recumbency. Necropsy of the hen revealed multifocal granulomatous pneumonia associated with inhaled dust, along with locally invasive follicular cysts on the wings, consistent with avian keratoacanthoma. The examination also revealed an overgrown, penetrating spur and peripheral nerve infiltrates, and avian keratoacanthoma, which may have contributed to clinical lameness, inability to move, and subsequent health deterioration.

IHC testing for MDV, ALV, and REV was negative, supporting the absence of known viral lymphoproliferative disease. Aerobic culture and antimicrobial susceptibility testing of a right-wing abscess yielded *Escherichia coli* (*E. coli*), *Pseudomonas aeruginosa* (*P. aeruginosa*), and *Proteus mirabilis* (*P. mirabilis*). Multidrug resistance was observed in the *P. aeruginosa* and *P. mirabilis* isolates, whereas the *E. coli* isolate was fully susceptible to all antimicrobials tested. A Gram-negative organism was additionally isolated from the liver. The colonies produced by this isolate were greyish on culture plates and appeared pleomorphic under microscopy. The bacterial colonies were pinpointed after overnight incubation but enlarged substantially after 72 h at 37 °C with 5% CO_2_ on 5% sheep blood agar. Growth occurred on chocolate agar but not on MacConkey agar. Two distinct colony morphotypes (CVDC-smooth and CVDC-rough) were observed (Supplementary Figure S1): a uniformly grey “smooth” variant (CVDC-smooth) and a “rough” variant with grey centers and translucent margins (CVDC-rough).

Initial identification using the VITEK^®^ 2 Gram-negative (GN) card classified both isolates as *Sphingomonas paucimobilis* with 90% probability; however, MALDI-TOF MS did not provide any identification. VITEK^®^ 2 GN card profiles were identical between the two morphotypes, despite their distinct colony morphology (Table 1). Both isolates were positive for D-glucose, sucrose, γ-glutamyl transferase, phosphatase, and arylamidases and negative for D-maltose, sugar alcohols, H_2_S production, urease, citrate and malonate utilization, and decarboxylases, lysine and ornithine decarboxylase activities. Both morphotypes were catalase-positive, oxidase-negative, and indole production-negative. These results demonstrated that routine phenotypic identification methods were insufficient for reliably identifying the isolate, prompting subsequent genomic characterization by whole-genome sequencing.

### 3.2. Read Quality and Genome Assembly and Annotation

SeqKit analysis of ONT and PacBio HiFi reads showed that N50 values ranged from 11,598 bp (PacBio HiFi) to 18,585 bp (ONT), with a mean GC content of ~49% (Table 2). AutoCycler assembly produced one large circular contig (CVDC-smooth: 2.18 Mb with ONT reads and 2.14 Mb with PacBio reads; CVDC-rough: 2.14 Mb with both ONT and PacBio reads) and a small contig of 2428 bp for each isolate. Assembly polishing with NextPolish improved base-level accuracy, as indicated by increased Merqury QV scores from 52.2 to 63.3 for CVDC-rough and from 57.1 to ≥63 for CVDC-smooth, with a slight increase in completeness (96.18–96.20% and 97.36–97.37%, respectively). BUSCO completeness scores were high (CVDC-smooth: 97.3%; CVDC-rough: 97.4%), and QUAST confirmed genome sizes and GC content, with both morphotypes exhibiting 49.15% GC. Self-alignment of ONT reads to the corresponding assemblies using the EPI2ME wf-bacterial-genome workflow yielded 100% primary read mapping for both isolates, indicating high read-to-assembly consistency. Genome annotation with Prokka identified 2108 coding sequences (CDSs) in CVDC-smooth and 2061 in CVDC-rough. Both genomes contained 19 rRNA genes, 59 tRNAs, and one tmRNA. The additional 47 CDSs in CVDC-smooth correspond to genes duplicated within the tandemly repeated ~20 kb genomic region. After accounting for overlapping CDSs at the repeat boundaries, the increase in CDS number was fully explained by the additional repeat copies rather than the acquisition of novel genes. The additional 2428 bp contig present in both isolates was examined separately by BLASTn and showed low nucleotide similarity to a *Neisseria gonorrhoeae* plasmid sequence (GenBank accession CP145022.1; 75.8% identity and 33% query coverage). Given the limited sequence similarity and coverage, the origin of this small contig could not be definitively determined. Overall, the combined ONT/PacBio workflow produced high-quality, near-complete chromosome-level genome assemblies suitable for downstream comparative genomic analyses.

### 3.3. Taxonomic Identification

Preliminary taxonomic identification of CVDC-smooth and CVDC-rough produced variable results across database-dependent classification tools. The Nextflow EPI2ME wf-bacterial-genome workflow classified both isolates as *Spirabiliibacterium mucosae*, with 96.4% ANI but only 15% reference genome coverage. BLASTn searches against the NCBI core_nt database instead returned *Avibacterium paragallinarum* as the closest match, although the alignment covered only 9% of the queried sequence and showed 83% nucleotide identity. PubMLST classified both isolates as *S. mucosae*, with 53% sequence identity, whereas Kraken2 did not assign a taxonomic classification. These inconsistent assignments, together with the low genome or sequence coverage observed for several database matches, limited the reliability of database-dependent tools for species-level identification. In contrast, GTDB-Tk placed both genomes within the genus *Spirabiliibacterium*, with *S. mucosae* as the closest reference (ANI, 93.2%; alignment fraction, 0.86–0.88), but did not assign either genome to an existing species because they fell outside the predefined ANI radius.

Additional sequence-comparison analyses provided evidence of relatedness to *S. mucosae* but did not independently establish species identity. BLASTn alignment of the *S. mucosae* type strain 20609/3T against the CVDC-smooth and CVDC-rough assemblies identified the largest contiguous alignment of approximately 74 kb with 94.7% nucleotide identity, representing approximately 3.4% of the reference genome. Minimap2 mapping showed that 91.4–97.6% of primary reads from the CVDC isolates aligned to the *S. mucosae* reference genome. Collectively, these findings indicated that both isolates were most closely associated with *Spirabiliibacterium*, but the database-dependent approaches alone were insufficient to resolve their taxonomic status. Genome-based comparative and phylogenetic analyses were therefore used to further assess taxonomy.

### 3.4. Phylogenetic and Comparative Genomic Analyses

**Figure 1:**
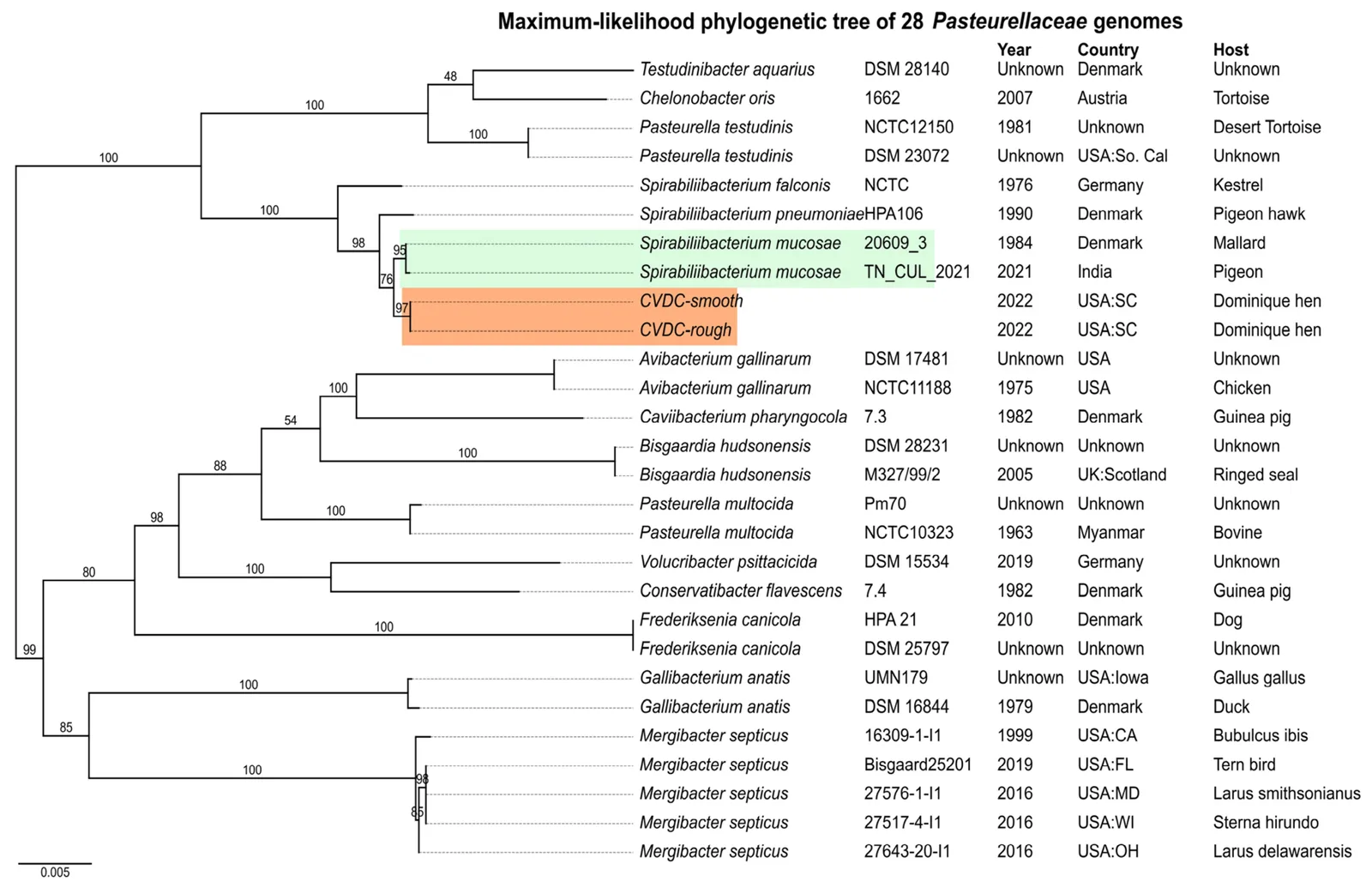
Maximum-likelihood phylogenetic tree of 28 *Pasteurellaceae* genomes. The tree illustrates the phylogenetic placement of CVDC-smooth and CVDC-rough (highlighted in orange), which form a distinct subclade within the *Spirabiliibacterium* lineage. These isolates share a common internal node with the *Spirabiliibacterium mucosae* subclade (shown in green), indicating a shared evolutionary history. Together with *S. pneumoniae* and *S. falconis*, these taxa form a well-supported monophyletic *Spirabiliibacterium* clade distinct from the Avibacterium gallinarum outgroup. Node values represent bootstrap support (%) based on 1000 replicates. Genome labels indicate strain designation, year of isolation, country of origin, and host source.

Phylogenetic analysis of 28 genomes revealed a well-supported monophyletic *Spirabiliibacterium* clade, clearly separated from *Avibacterium gallinarum* (bootstrap 95–100; Figure 1). The CVDC-smooth and CVDC-rough isolates clustered closely with *S. mucosae*, forming a distinct subclade within the *Spirabiliibacterium* lineage. Pairwise ANI analysis showed 100% identity between CVDC-smooth and CVDC-rough, but approximately 93% identity relative to two *S. mucosae* strains, below the commonly accepted 95% ANI species-level threshold [43]. Digital DNA–DNA hybridization (dDDH) values were 52%, with only a 24.96% probability that the true dDDH value exceeds the 70% species-level threshold, further supporting genomic divergence from the currently described *S. mucosae* strains [44,45,46]. Additionally, SNP distance analysis revealed no SNP differences between the CVDC-smooth and CVDC-rough (Table 3), indicating they are genomically identical within shared alignment regions. In contrast, comparisons between the CVDC isolates and the available *Spirabiliibacterium* reference genomes identified more than 4000 SNP differences (Table 3), providing additional evidence of substantial genomic divergence. Together with their distinct phylogenetic placement within the *Spirabiliibacterium* clade, the ANI, dDDH, and SNP analyses support the interpretation that CVDC-smooth and CVDC-rough are clonal variants of a genomically distinct *Spirabiliibacterium* lineage closely related to *S. mucosae*. Although the ANI and dDDH values are consistent with species-level genomic divergence from currently described *S. mucosae*, we conservatively refer to CVDC-smooth and CVDC-rough as a genomically distinct *Spirabiliibacterium* lineage, as formal description and nomenclatural proposal of a novel species were beyond the scope of this study.

### 3.5. Pan-Genome Structure, Virulence and AMR Profiles

Roary analysis of the 28-genome dataset identified 32,617 gene clusters, including 16 core genes and 1 soft-core gene. Scoary2 identified 312 gene clusters associated with the six *Spirabiliibacterium* genomes relative to the other *Pasteurellaceae* genomes included in the analysis (Table S3). Lineage-specific comparisons showed 377 gene clusters associated with *S. mucosae* (Table S4), 732 gene clusters shared among CVDC-smooth, CVDC-rough, and *S. mucosae* genomes (Table S5), and 543 gene clusters associated with CVDC-smooth and CVDC-rough isolates (Table S6). Although distinct gene presence–absence patterns were observed for the *S. mucosae* and CVDC groups, these associations did not remain statistically significant after multiple-testing correction and should therefore be interpreted as exploratory.

**Figure 2:**
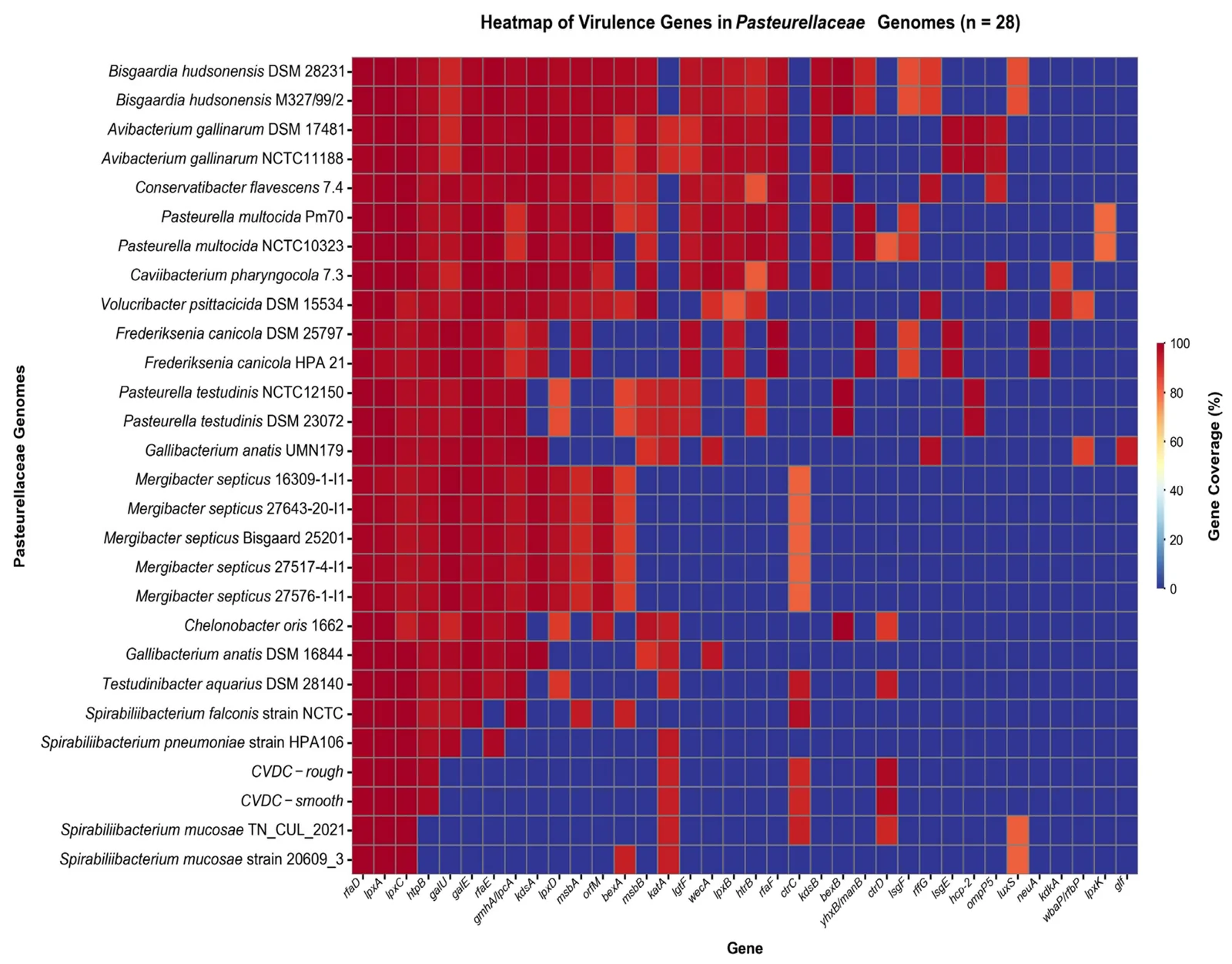
Heatmap generated using a custom Python script showing the distribution of virulence-associated genes identified using ABRicate (VFDB) across 28 *Pasteurellaceae* genomes. *Spirabiliibacterium mucosae* strains and the isolates CVDC-smooth and CVDC-rough share six conserved genes (*rfaD*, *lpxA*, *lpxC*, *katA*, *ctrC*, and *ctrD*). The gene *htpB* is present in all genomes except *S. mucosae*, whereas three genes (*rfaD*, *lpxA*, and *lpxC*) are conserved across all 28 *Pasteurellaceae* genomes. Virulence-associated genes are shown on the x-axis, and the analyzed genomes are shown on the y-axis. Heatmap intensity represents gene coverage, ranging from 0% to 100%, with 0% indicating the absence of a detectable homolog.

Virulence gene screening using VFDB (Figure 2) identified three conserved genes (*rfaD*, *lpxA*, *lpxC*) across all 28 genomes. The CVDC-smooth and CVDC-rough and the two *S. mucosae* strains shared six virulence-associated genes (*rfaD*, *lpxA*, *lpxC*, *katA*, *ctrC*, and *ctrD*). The heat shock protein gene *htpB* was present in all the analyzed genomes except those of the *S. mucosae* strains. The AMR gene screening with ResFinder and ABRicate detected no acquired resistance genes in either CVDC isolate, whereas CARD identified limited intrinsic determinants, including penicillin-binding protein variants, elfamycin markers, and SMR-family efflux pumps. Based on the surrogate reference criteria used during the diagnostic evaluation, disk-diffusion profiles were consistent with susceptibility to the tested antimicrobials, except streptomycin, which showed a profile interpreted as resistant using the reference criteria available to the diagnostic laboratory. Because validated interpretive breakpoints are unavailable for *Spirabiliibacterium*, these findings are presented as descriptive phenotypic observations rather than formal susceptibility classifications. Overall, the CVDC isolates exhibited a limited repertoire of detected virulence-associated and antimicrobial resistance genes, consistent with their close genomic relationship to *S. mucosae*.

### 3.6. Variant Calling and Tandem Repeat Analysis of CVDC-Smooth and CVDC-Rough

Variant calling of ONT and PacBio HiFi reads from CVDC-rough generated identical profiles; consequently, PacBio HiFi-derived variants were reported. No single-nucleotide substitutions were detected between CVDC-smooth and CVDC-rough. Six localized insertion–deletion variants were identified (Table S7), comprising five intergenic variants and one coding insertion within the nucleoside transporter gene *nupC*. All variants exhibited high variant fractions (0.78–0.99), indicating reliable, fixed mutations.

**Figure 3:**
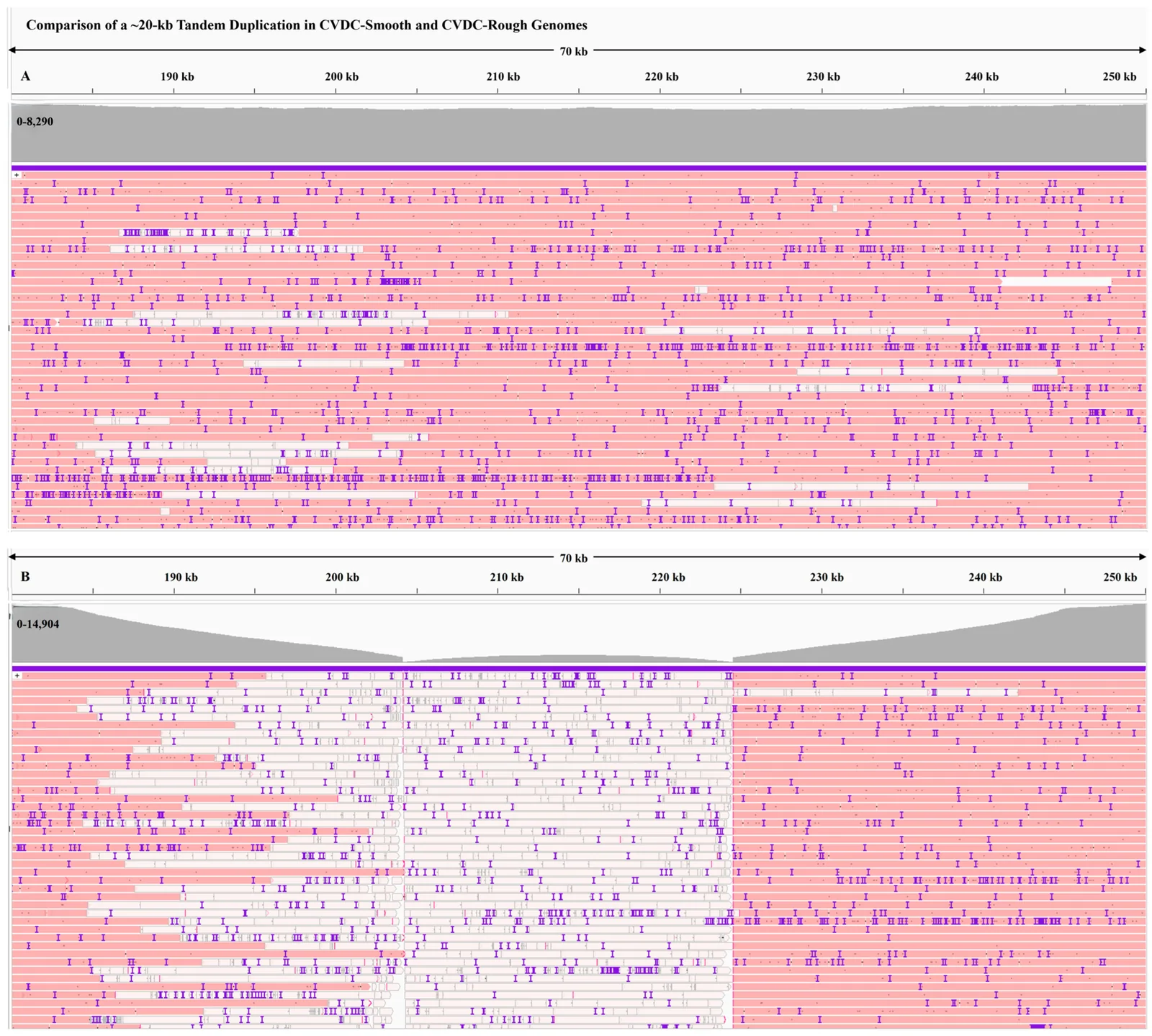
Structural variation analysis of CVDC-smooth and CVDC-rough genomes. (**A**). CVDC-smooth shows a tandem duplication of three ~20 kb repeat units supported by long ONT reads, uniform coverage, and continuous flanking alignment. (**B**) CVDC-rough contains a single copy of this region, with uneven coverage, gaps at repeat boundaries, and the lack of long-read support, consistent with a single-copy configuration.

Despite the absence of core-genome SNPs, a 40 kb size difference was observed between CVDC-smooth and CVDC-rough genomes. Structural variation analysis using Minimap2 and IGV identified a prominent tandem duplication in CVDC-smooth, comprising three consecutive ~20 kb repeat units spanning positions 183,749–244,952 bp (Figure 3A). This configuration was supported by long ONT reads spanning the entire repeat array, uniform read depth across the duplicated region, and uninterrupted coverage at the flanking boundaries. In contrast, the corresponding region in CVDC-rough lacked long-read support and was represented only by short-read alignments, which showed uneven coverage, zero mapping quality, and gaps at the repeat boundaries, consistent with a single-copy configuration. Accordingly, the corresponding genomic region was present as a single copy in CVDC-rough (positions 334,915–355,320 bp) (Figure 3B). Although PacBio HiFi reads (~11 kb) were insufficient to span the full repeat array in CVDC-smooth, alignments demonstrated continuous coverage across the flanking regions. In CVDC-rough, HiFi alignments contained gaps across the repeat-region junctions, further supporting its single-copy status. BLASTn comparisons confirmed that the repeat expansion was unique to CVDC-smooth and absent from CVDC-rough and two strains of S. mucosae, all of which harbored a single-copy version of this region. The ~20 kb repeat region encodes genes involved in metabolism, transport, and stress response, including representative genes such as *frdA-D*, *pykA*, *modA-E*, *btuD*, *pspB-C*, the *Sap A-D* and *SapF* transporter system, and the membrane-associated enzyme *uppP*. Collectively, these findings indicate that copy-number variation within the tandemly duplicated ~20 kb region represents the primary genomic difference between the CVDC-smooth and CVDC-rough morphotypes and may contribute to their distinct colony phenotype.

## 4. Discussion

This study describes the isolation and characterization of two phenotypically distinct but genomically closely related bacterial isolates recovered from the liver of a backyard hen with multifactorial disease. The bird had concurrent disease conditions, including granulomatous pneumonia, keratoacanthoma, overgrown penetrating spur and peripheral nerve infiltrates, which may have compromised host defenses and facilitated opportunistic invasion. Members of the family *Pasteurellaceae* include both primary pathogens and opportunistic colonizers in avian hosts, particularly in immunocompromised or stressed individuals [1,8,9]. In this context, recovery of an unclassified Gram-negative organism from a normally sterile site warranted further investigation.

Phenotypically, CVDC-smooth and CVDC-rough displayed features consistent with *S. mucosae*, including growth on blood and chocolate agar but not MacConkey agar, pleomorphism, and a catalase-positive, oxidase-negative, indole-negative profile [17]. Despite distinct colony morphologies, basic biochemical testing and VITEK 2 profiles were indistinguishable between CVDC-smooth and CVDC-rough, indicating that the observed phenotypic variation is not captured by standard metabolic assays. Both the basic and Vitek2 card biochemical profiles were broadly comparable to those of *S. mucosae*, with minor lineage-specific variations. In particular, β-glucosidase activity, coumarate utilization, O/129 resistance, and Ellman reactions were positive in *S. mucosae* but negative in CVDC-smooth and CVDC-rough isolates (Table S8) [10,17]. A notable finding was the strong concordance between observed phenotypes and genome-derived metabolic predictions. Catalase positivity, oxidase negativity, and indole negativity were consistent with the presence of *katA* [56] and the absence of cytochrome c oxidase components [57] and *tnaA* [58], respectively, supporting the reliability of genome-based metabolic inference for poorly represented taxa and strengthening confidence in the taxonomic placement of both CVDC-smooth and CVDC-rough within *Spirabiliibacterium* [56,57,58]. Despite this overall agreement, VITEK^®^ 2 misidentified the organism, and MALDI-TOF MS failed to assign its taxonomy, highlighting the limitations of database-dependent systems for uncommon taxa, consistent with previous reports of *S. mucosae* misidentified as *Burkholderia mallei* [12,13,17]. Similar diagnostic challenges have been reported for other uncommon bacterial taxa, where routine phenotypic methods were unable to provide reliable identification and whole-genome sequencing was ultimately required to resolve taxonomic identity [18]. In the present study, whole-genome sequencing (WGS) provided high-resolution taxonomic placement and further demonstrates its value for identifying unusual bacterial isolates in diagnostic settings [17,45,46]. These findings also emphasize the importance of expanding MALDI-TOF MS and automated biochemical reference databases to improve the identification of uncommon and underrepresented veterinary bacterial taxa.

High-quality hybrid assemblies generated using ONT and PacBio HiFi reads yielded near-complete genomes suitable for comparative analysis. Database-driven taxonomic tools produced inconsistent classifications, reflecting the limited representation of *Spirabiliibacterium* in public repositories. However, genome-based methods consistently placed both CVDC-smooth and CVDC-rough within this genus. Average nucleotide identity (~93%) and digital DNA–DNA hybridization (~52%) [45,46] values relative to *S. mucosae* fell below accepted species-delineation thresholds, indicating that both CVDC-smooth and CVDC-rough represent a distinct lineage within *Spirabiliibacterium*. Phylogenetic analysis further supported placement of the isolates within the genus *Spirabiliibacterium* [10,17]. Previously reported *Spirabiliibacterium* isolates were recovered from pigeons and ducks, whereas the isolate described in this study was obtained from the liver of a backyard chicken. This finding expands the known avian host range of the genus and, to our knowledge, represents the first report of a *Spirabiliibacterium* lineage recovered from a chicken in the United States. Consistent with the ANI and dDDH analyses, SNP distance analysis identified 4193 SNP differences between CVDC-smooth and CVDC-rough and *S. mucosae* genomes, supporting their recognition as a genomically distinct lineage within the genus *Spirabiliibacterium* [17,59]. In contrast, SNP distance analysis showed no differences between the smooth and rough morphotypes, indicating that both represent the same lineage rather than separate strains [17,59]. Although genomic analyses indicate that the CVDC-smooth and CVDC-rough isolates are distinct from the currently described *S. mucosae* strains, the objective of this study was not to formally describe a new species but rather to characterize a genomically distinct *Spirabiliibacterium* lineage recovered from a chicken. To facilitate future taxonomic, comparative genomic, and phenotypic investigations, deposition of a representative isolate in a public culture collection is currently in progress.

The family-wide pan-genome analysis revealed substantial gene-content diversity across the analyzed *Pasteurellaceae*, consistent with the genomic plasticity described for this family [17,22]. The small number of core genes likely reflects, at least in part, the broad phylogenetic diversity of the 28-genome dataset. Gene-content comparisons identified patterns associated with the six analyzed *Spirabiliibacterium* genomes, as well as differences in gene content between the CVDC isolates and *S. mucosae*. However, some lineage-level associations did not remain statistically significant after multiple-testing correction and should be considered exploratory, particularly given the limited number of *Spirabiliibacterium* genomes and the phylogenetic structure of the dataset [17]. Additional genomes will be needed to more comprehensively characterize gene-content diversity at the genus and lineage levels.

Comparative analysis of 28 *Pasteurellaceae* genomes showed that *Spirabiliibacterium* genomes harbored fewer VFDB-matched virulence-associated genes than other genera included in the analysis. This finding should be interpreted in the context of database-dependent detection, as limited representation of *Spirabiliibacterium*-specific virulence determinants in VFDB and sequence divergence may reduce the detection of homologs and therefore does not necessarily indicate a lower overall virulence potential. VFDB analysis showed that both CVDC-smooth and CVDC-rough carried seven virulence-associated genes, including the three genes (*rfaD*, *lpxA*, and *lpxC*) conserved across all 28 *Pasteurellaceae* genomes [60]. Specifically, *lpxA* and *lpxC* participate in lipid A biosynthesis, a core component of lipopolysaccharide [61]. The gene *rfaD* contributes to the formation of the LPS core oligosaccharide [62]. The capsular polysaccharide transport genes *ctrC* and *ctrD* [63,64], support cell surface integrity, and *htpB* [65] encodes a heat shock chaperone involved in protein folding under stress conditions. Database-dependent variation was also observed for *luxS*, a gene involved in quorum sensing and biofilm formation [17,66]. VFDB detected the *luxS* gene only in *S. mucosae* strains, whereas genome annotation pipelines identified it in CVDC-smooth and CVDC-rough [17]. To resolve this discrepancy, we further verified the predicted *LuxS* protein using NCBI BLASTp, which showed 97% amino acid identity and 100% query coverage to *LuxS* from *S. mucosae*. These findings support the presence of *luxS* in the CVDC isolates and suggest that its non-detection by VFDB likely reflects differences in database representation, sequence divergence, and homology-based detection thresholds rather than true biological absence. AMR determinants were sparse in CVDC-smooth and CVDC-rough, consistent with disk-diffusion profiles suggestive of susceptibility according to the surrogate reference criteria used during the diagnostic evaluation; however, these findings should be interpreted with caution because validated breakpoints are unavailable for *Spirabiliibacterium*. As previously reported for the *S. mucosae* type strain 20609/3ᵀ, the tetracycline resistance gene *tet*(*B*) was not detected in the CVDC genomes [17], suggesting that this resistance determinant is not uniformly distributed within this broader clade. In contrast, *tet*(*B*) gene has been reported in *S. mucosae* strain TN_CUL_2021 [17]. Streptomycin showed a profile interpreted as resistant according to the surrogate reference criteria, despite the absence of known resistance genes, suggesting a potential intrinsic or uncharacterized mechanism [67]; however, this observation requires further investigation. Given the limited phenotypic antimicrobial susceptibility data available for *Spirabiliibacterium*, these preliminary observations may serve as a useful reference for future studies.

Despite limited variation in virulence and AMR profiles, CVDC-smooth and CVDC-rough were highly similar at the nucleotide level, with only a small number of INDELs detected in the CVDC-rough relative to the CVDC-smooth. The major genomic difference was a three-copy expansion of an ~20 kb region in CVDC-smooth, whereas CVDC-rough and the two strains of *S. mucosae* retained a single copy. Long-read mapping and uniform coverage across the duplicated region confirmed that this expansion represents a genuine genomic feature rather than an assembly artifact. The duplicated locus encodes genes involved in metabolism, membrane integrity, and stress response, and increased gene dosage provides a plausible mechanism for variation in colony morphology between the two morphotypes [68]. Despite differences in genome size, the absence of core genome SNPs, the presence of only a few small indels, and 100% ANI indicate that the morphotypes represent clonal variants rather than distinct strains. However, the observed association between repeat copy number and colony morphology does not establish causality, and functional validation will be required to determine whether the tandem duplication directly contributes to the phenotypic difference. Further studies are needed to determine the functional impact of this tandem duplication. In addition, investigations into the prevalence, host range, and clinical significance of this *Spirabiliibacterium* lineage in poultry and other avian species will improve our understanding of its epidemiology and biological significance.

The clinical significance of this organism remains uncertain. The concurrent disease processes may have compromised host defenses and provided a context for opportunistic recovery of the organism from the liver, consistent with the opportunistic behavior described for other members of the *Pasteurellaceae* [1,69]. However, the single-case nature of this study precludes conclusions regarding pathogenicity or systemic dissemination. This study expands the known diversity of *Spirabiliibacterium* and identifies a genomically distinct lineage associated with poultry. Structural genomic variation, rather than SNP-level divergence, appears to underlie phenotypic heterogeneity within this lineage. Collectively, these findings highlight limitations of conventional diagnostic methods and support the use of whole-genome sequencing for accurate identification and characterization of unusual bacterial isolates in veterinary diagnostics.

## Figures and Tables

**Table 1:** VITEK^®^ 2 Gram-negative (GN) card biochemical profiles of CVDC-smooth and CVDC-rough isolates.

Well	Test	Mnemonic	CVDC-Smooth	CVDC-Rough
2	Ala-Phe-Pro-ARYLAMIDASE	APPA	−	−
3	ADONITOL	ADO	−	−
4	L-Pyrrolydonyl-ARYLAMIDASE	PyrA	−	−
5	L-ARABITOL	IARL	−	−
7	D-CELLOBIOSE	dCEL	−	−
9	BETA-GALACTOSIDASE	BGAL	−	−
10	H2S PRODUCTION	H2S	−	−
11	BETA-N-ACETYL-GLUCOSAMINIDASE	BNAG	−	−
12	Glutamyl Arylamidase pNA	AGLTp	−	−
13	D-GLUCOSE	dGLU	+	+
14	GAMMA-GLUTAMYL-TRANSFERASE	GGT	+	+
15	FERMENTATION/GLUCOSE	OFF	−	−
17	BETA-GLUCOSIDASE	BGLU	−	−
18	D-MALTOSE	dMAL	−	−
19	D-MANNITOL	dMAN	−	−
20	D-MANNOSE	dMNE	−	−
21	BETA-XYLOSIDASE	BXYL	−	−
22	BETA-Alanine arylamidase pNA	BAIap	−	−
23	L-Proline ARYLAMIDASE	ProA	+	+
26	LIPASE	LIP	−	−
27	PALATINOSE	PLE	−	−
29	Tyrosine ARYLAMIDASE	TyrA	+	+
31	UREASE	URE	−	−
32	D-SORBITOL	dSOR	−	−
33	SACCHAROSE/SUCROSE	SAC	+	+
34	D-TAGATOSE	dTAG	−	−
35	D-TREHALOSE	dTRE	−	−
36	CITRATE (SODIUM)	CIT	−	−
37	MALONATE	MNT	−	−
39	5-KETO-D-GLUCONATE	5KG	−	−
40	L-LACTATE alkalinization	ILATk	−	−
41	ALPHA-GLUCOSIDASE	AGLU	−	−
42	SUCCINATE alkalinization	SUCT	−	−
43	Beta-N-ACETYL-GALACTOSAMINIDASE	NAGA	−	−
44	ALPHA-GALACTOSIDASE	AGAL	−	−
45	PHOSPHATASE	PHOS	+	+
46	Glycine ARYLAMIDASE	GlyA	+	+
47	ORNITHINE DECARBOXYLASE	ODC	−	−
48	LYSINE DECARBOXYLASE	LDC	−	−
53	L-HISTIDINE assimilation	IHISa	−	−
56	COUMARATE	CMT	−	−
57	BETA-GLUCURONIDASE	BGUR	−	−
58	O/129 RESISTANCE (comp. vibrio.)	O129R	−	−
59	Glu-Gly-Arg-ARYLAMIDASE	GGAA	−	−
61	L-MALATE assimilation	IMLTa	(−)	−
62	ELLMAN	ELLM	−	−
64	L-LACTATE assimilation	ILATa	−	−

**Table 2:** Seqkit-derived quality metrics for sequencing reads from CVDC-smooth and CVDC-rough.

Parameter	CVDC-Rough	CVDC-Rough	CVDC-Smooth	CVDC-Smooth
	PacBio	ONT	PacBio	ONT
Format	FASTQ	FASTQ	FASTQ	FASTQ
Type	DNA	DNA	DNA	DNA
Num_seqs	448,313	3,860,174	488,711	2,629,437
Sum_len	5,263,368,463	34,387,493,888	6,101,321,108	20,028,319,930
Min_len	270	5	148	5
Avg_len	11,740.4	8908.3	12,484.5	7617
Max_len	44,430	1,121,731	46,772	808,968
Q1	10,225	1462	10,518	958
Q2	11,216	4863	11,817	3977
Q3	12,807	12,084	13,826	9332
Sum_gap	0	0	0	0
N50	11,598	18,585	12,375	16,396
N50_num	10,924	49,631	12,071	52,989
Q20 (%)	98.15	91.52	98.02	89.95
Q30 (%)	95.67	85.13	95.34	82.37
AvgQual	26.3	19.41	26.03	18.78
GC (%)	49.09	49.1	49.11	49.08

**Table 3:** Pairwise single-nucleotide polymorphism (SNP) distances between CVDC isolates and *Spirabiliibacterium* genomes.

Sample	*S. mucosae* TN_CUL_2021	*S. falconis* Strain NCTC	*S. mucosae* Strain 20609/3	CVDC-Rough	CVDC-Smooth	*S. pneumoniae* Strain HPA106
*S. mucosae* TN_CUL_2021	0	10,432	749	4254	4254	7864
*S. falconis* strain NCTC	10,432	0	10,254	11,574	11,574	11,802
*S. mucosae* strain 20609/3	749	10,254	0	4193	4193	7723
CVDC-rough	4254	11,574	4193	0	0	9020
CVDC-smooth	4254	11,574	4193	0	0	9019
*S. pneumoniae* strain HPA106	7864	11,802	7723	9020	9019	0

## Data Availability

Genome assemblies and raw sequencing reads generated in this study have been deposited in the National Center for Biotechnology Information (NCBI) under BioProject accessions PRJNA1447504 and PRJNA1448032. The isolates CVDC-smooth and CVDC-rough are associated with BioSample accessions SAMN56998712 and SAMN56998713, respectively. The genome accession numbers were JCCHDS000000000 for the CVDC-rough isolate, and JCCHDT000000000 for the CVDC-smooth isolate. 16S rRNA gene sequences for both isolates have been deposited in NCBI GenBank, with accession numbers PZ459834 and PZ459835. All software tools, versions, and parameters used in this study were provided in Supplementary Table S2. Deposition of a representative isolate in a public culture collection is currently in progress, and the accession number will be provided once available. Additional data supporting the findings of this study are available from the corresponding author upon reasonable request.

## Supplementary Materials

The following supporting information can be downloaded at https://www.mdpi.com/article/10.3390/microorganisms14091953/s1, Supplementary Figure S1: Colonies of CVDC-smooth (A) and CVDC-rough (B) bacterial isolates. Supplementary Figure S2: Comparison of a ~20-kb Tandem Duplication in CVDC-Smooth and CVDC-Rough Genomes. Table S1: NCBI Genome accession numbers used for phylogenetic and comparative genomic analyses; Table S2: Bioinformatics tools, versions, and parameters used in this study; Table S3: Scoary2 identified 312 gene clusters associated with six *Spirabiliibacterium* genomes; Table S4: Gene presence–absence patterns in *Spirabiliibacterium mucosae* identified by Scoary2 pangenome analysis; Table S5: Shared gene content among CVDC-smooth, CVD-rough, and *Spirabiliibacterium mucosae* based on Scoary2 pangenome analysis; Table S6: Gene presence–absence patterns associated with CVDC-smooth and CVDC-rough isolates within *Pasteurellaceae* identified by Scoary2 pangenome analysis; Table S7: High-confidence insertion–deletion variants between CVDC-smooth and CVDC-rough isolates; Table S8: Biochemical characteristics of CVDC-smooth and CVDC-rough compared with published *Spirabiliibacterium mucosae*. A custom Python script for phylogenetic tree visualization and heatmap generation will be provided upon request.
